# Protease-Activated Receptor 2 Mediates Mucus Secretion in the Airway Submucosal Gland

**DOI:** 10.1371/journal.pone.0043188

**Published:** 2012-08-15

**Authors:** Hyun Jae Lee, Yu-Mi Yang, Kyubo Kim, Dong Min Shin, Joo-Heon Yoon, Hyung-Ju Cho, Jae Young Choi

**Affiliations:** 1 Brain Korea 21 Project for Medical Science, Yonsei University College of Medicine, Seoul, Korea; 2 Department of Otorhinolaryngology, Yonsei University College of Medicine, Seoul, Korea; 3 Airway Mucus Institute, Yonsei University College of Medicine, Seoul, Korea; 4 Research Center for Human Natural Defense System, Yonsei University College of Medicine, Seoul, Korea; 5 Department of Oral Biology, Brain Korea 21 Project, Oral Science Research Center, Yonsei University College of Dentistry, Seoul, Korea; 6 Department of Otorhinolaryngology, Kang-Dong Sacred Heart Hospital, Hallym University College of Medicine, Seoul, Korea; Children’s Hospital Los Angeles, United States of America

## Abstract

Protease-activated receptor 2 (PAR2), a G protein-coupled receptor expressed in airway epithelia and smooth muscle, plays an important role in airway inflammation. In this study, we demonstrated that activation of PAR2 induces mucus secretion from the human airway gland and examined the underlying mechanism using the porcine and murine airway glands. The mucosa with underlying submucosal glands were dissected from the cartilage of tissues, pinned with the mucosal side up at the gas/bath solution interface of a physiological chamber, and covered with oil so that secretions from individual glands could be visualized as spherical bubbles in the oil. Secretion rates were determined by optical monitoring of the bubble diameter. The Ca^2+^-sensitive dye Fura2-AM was used to determine intracellular Ca^2+^ concentration ([Ca^2+^]_i_) by means of spectrofluorometry. Stimulation of human tracheal mucosa with PAR2-activating peptide (PAR2-AP) elevated intracellular Ca^2+^ and induced glandular secretion equal to approximately 30% of the carbachol response in the human airway. Porcine gland tissue was more sensitive to PAR2-AP, and this response was dependent on Ca^2+^ and anion secretion. When the mouse trachea were exposed to PAR2-AP, large amounts of secretion were observed in both wild type and ΔF508 cystic fibrosis transmembrane conductance regulator mutant mice but there is no secretion from PAR-2 knock out mice. In conclusion, PAR2-AP is an agonist for mucus secretion from the airway gland that is Ca^2+^-dependent and cystic fibrosis transmembrane conductance regulator-independent.

## Introduction

Airway submucosal glands produce most of the airway mucus, which is essential for mucociliary clearance. The submucosal gland also secretes various antimicrobial components, such as lysozyme, to protect the airway from bacteria [1]. Adequate mucus secretion from airway submucosal glands is essential to maintain the airway defense system. Defective mucus secretion may result in failure of host defense against pathogens, which in turn could be the underlying pathogenesis of airway infection in patients with cystic fibrosis (CF) [2]. In contrast, overproduction of mucus secretion from airway glands may lead to airway diseases, such as chronic obstructive lung disease and asthma [3]. Thus, tight control of mucus secretion is critical. Secretion from airway glands is mainly controlled by central parasympathetic input [4]. In addition to the autonomic nervous system, airways have abundant intrinsic neurons and pathogen-sensing receptors, and their activation induces mucus secretion from airway glands via a neuronal mediator such as substance P or vasoactive intestinal peptide (VIP) [2], [5]. There is accumulating evidence supporting an important role for these local reflexes in the airway innate immune response.

Protease-activated receptors (PARs) are G protein-coupled receptors that are activated by proteolytic cleavage of the N-terminal extracellular domain, leading to intracellular Ca^2+^ elevation [6], [7]. PARs have a variety of biologic roles and are involved in inflammatory diseases, including inflammatory bowel disease and rheumatoid arthritis [8]. PARs are expressed in airway epithelia [8], [9] and play an important role in inflammation and adaptive immunity by regulating functional responses of immune cells [10]. Endogenous PAR activators such as mast cell tryptase and neutrophil elastase induce airway inflammation and immune responses [11], and microorganism-derived proteases such as house dust mite allergens are also capable of activating PARs and inducing the release of pro-inflammatory cytokines from airway epithelial cells [12], [13]. More interestingly, a bacterial protease has been found to disable PARs and inhibit PAR-triggered signaling in airway epithelial cells [14]. Thus, PARs are an integral component of the airway defense system and may reveal the exact pathway by which proteases affect innate immune responses. However, the role of PARs in the innate immune system in the human airway under physiological and pathophysiological conditions remains unclear.

Among the various subtypes, PAR2 plays a major role in ion transport and fluid secretion from airway epithelial cell cultures. PAR2 activates the Ca^2+^-activated Cl^−^ channel (CaCC) in human bronchial epithelial cell lines and the mouse trachea [15]. PAR2 also induces a transepithelial current through the cystic fibrosis transmembrane conductance regulator (CFTR) by cytosolic Ca^2+^ mobilization in Calu-3 cells [16], [17]. Miotto *et al.* reported that PAR2 is also expressed in airway glands [18]. These findings suggest that PAR2 may regulate anion and fluid secretion in the airway submucosal gland. In contrast to its known function in ion transport and fluid secretion, the role of PAR2 in airway mucus secretion remains controversial as PAR2-activating peptide (PAR2-AP) is unable to induce mucin production in NCI-H292 cells [18] is only a weak enhancer of mucin secretion in human bronchial epithelial cells [19]. However, until now there has been no evidence that a PAR is involved in mucus secretion from the airway submucosal gland. Therefore, demonstrating the role of PAR2 in mucus secretion from the airway submucosal gland will provide a better understanding of the host defense system in the airway.

In this study, we show that activation of PAR2 in the human airway gland induces mucus secretion and we dissect the underlying mechanism in porcine and murine airway glands.

## Results

### PAR2-AP-induced Mucus Secretion in Human Airway Glands

**Figure 1:**
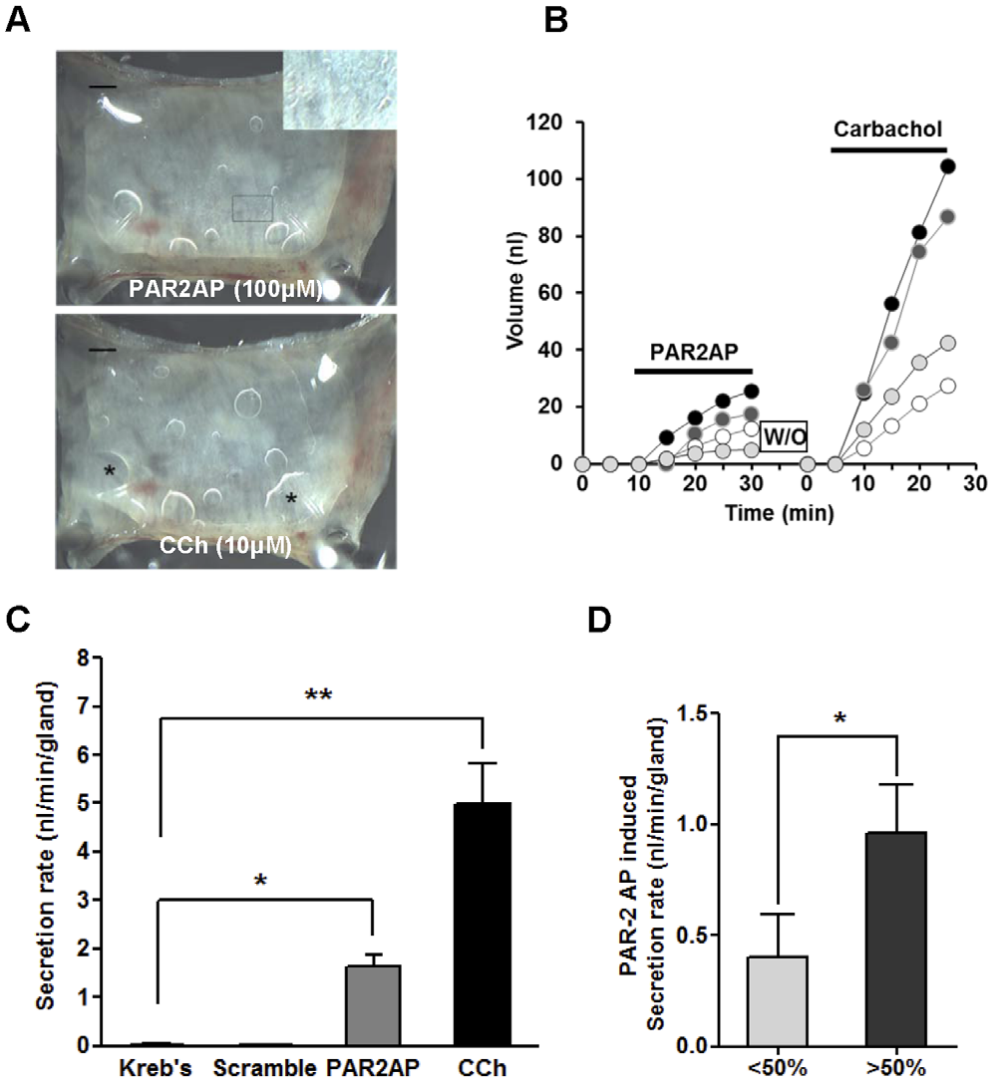
Serosal PAR2-AP stimulates mucus secretion from human airway submucosal glands. (A) Images of mucus bubbles formed under oil at the orifices of single submucosal glands 15 min after PAR2-AP (100 µM) or carbachol (10 µM) stimulation. PAR2-AP also induced small bubbles on the mucosal surface which seem to have originated from the epithelial surface inset of (A). * indicates the merging of several bubbles (*). Scale bar: 0.5 mm. **(**B) Plots of secreted mucus volume over time for four individual glands are shown. Each line represents a single gland. (C) Summary data of 52 glands from 11 subjects showing average secretion rates (± S.E.M.) for 20-min periods following application of PAR2-AP (100 µM) and carbachol (10 µM). Scrambled peptide did not induce mucus secretion. (D) PAR-2 AP induced mucus secretion according to the level of PAR2 expression. * and ** indicate significant differences from the control, *P*<0.05 and 0.005, respectively.

Serosal application of PAR2-AP (100 µM) to human tracheal mucosa markedly increased glandular secretion, resulting in bubble formation that was comparable to the response achieved with carbachol (10 µM), a potent cholinergic agonist. Along with the bubbles from submucosal glands, PAR2 stimulation induced tiny bubbles which appeared to originate from the surface of the epithelial cells (Figure 1A). A plot of mucus volume versus time for four individual glands from a single subject is shown in Figure 1B. PAR2-AP produced a short-latency transient peak, followed by sustained secretion of more than 20 min. The secretion rate varied among the glands but all the carbachol-responding glands were also activated by PAR2-AP. Summarized data from 11 human subjects are shown in Figure 1C. The mean secretion rate for the first 20 min of PAR2-AP treatment (100 µM) was 1.63±0.2 nl/min, which is approximately 30% of the response achieved with 10 µM carbachol (4.99±0.8 nl/min). As a next, we compared the PAR2-AP induced secretion rate according to the level of PAR-2 expression by immunostaining. The PAR2-AP induced mucus secretion is much bigger in tissues where the PAR-2 is expressed in >50% of acinar cells (1.08±0.5 nl/min) than in tissues the expression is <50% of acinar cells (0.37±0.6 nl/min). (Figure 1D).

### PAR2-AP-induced [Ca^2+^]_i_ Mobilization and the Localization of PAR2 in the Human Airway Gland

**Figure 2:**
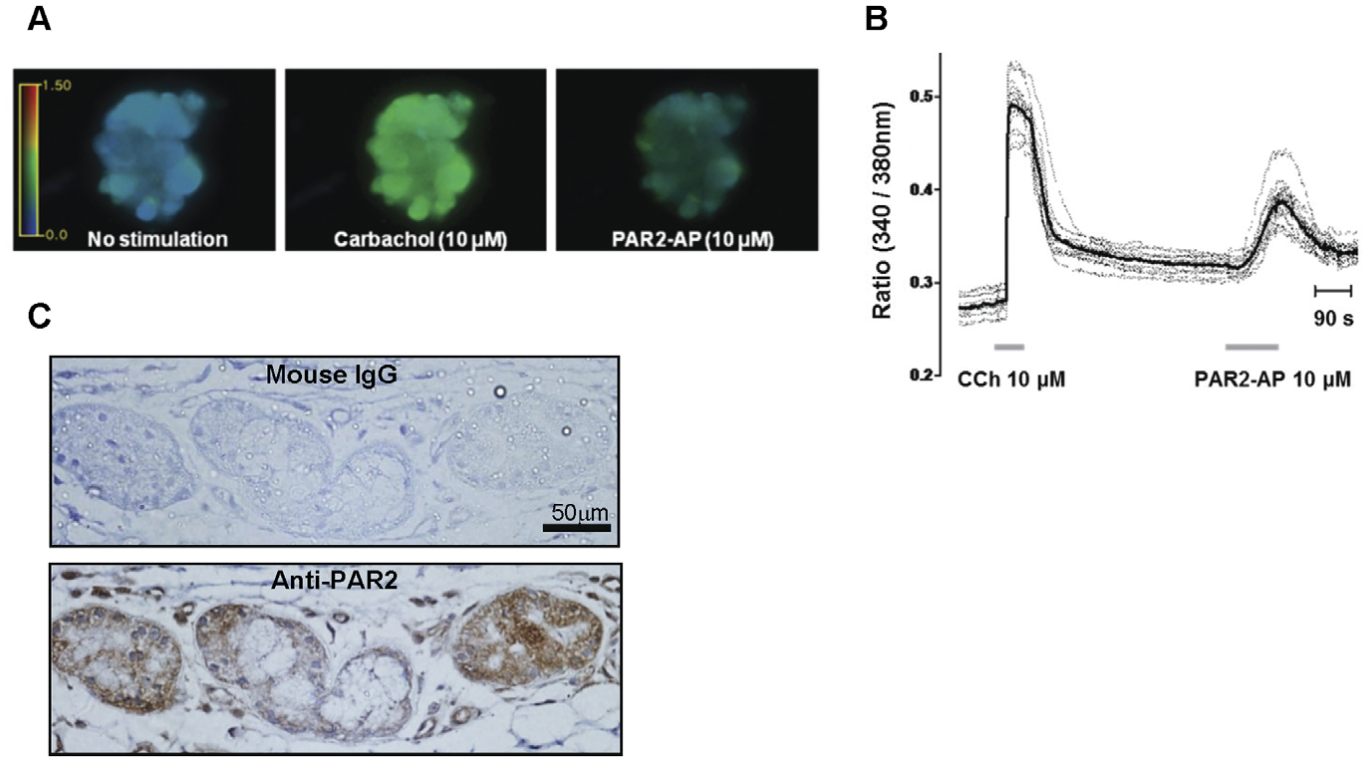
PAR2-AP induced [Ca^2+^]_i_ changes and PAR2 expression in human airway submucosal glands. (A) Fluorescence changes in response to 10 µM carbachol and 10 µM PAR2-AP. Cell diameters in images are approximately 20 µm. (B) [Ca^2+^]_i_ vs. time for 11 cells from images in (A), measured in response to sequential pulses of 10 µM carbachol or 10 µM PAR2-AP. Fluorescence ratio, 340 nm/380 nm. (C) Immunostaining using PAR2 antibody showed that PAR2 is mainly expressed on the basolateral side of acinar cells. In serous glandular cells, immunoreactivity is also noted in the cytoplasm. The negative control with mouse IgG shows no immunoreactivity. Scale bar: 50 µm.

We examined the changes in [Ca^2+^]_i_ after PAR2-AP application in dissected human airway gland cells. Because cells were found to be more sensitive to PAR2-AP than gland tissue, only 10 µM PAR2-AP was used. A slow increase in [Ca^2+^]_i_ was evoked by PAR2-AP within 100 sec, followed by a small sustained plateau. Although the response was not uniform among the cells, we observed similar responses. The peak response was approximately 40% of that seen with carbachol treatment (10 µM; Figure 2A,B). Immunostaining for PAR2 revealed that PAR2 proteins are expressed on the basolateral side of acinar cells of the submucosal gland. Immunoreactivity was also noted in the cytoplasm of acinar serous but not mucous cells (Figure 2C).

### PAR2-AP-induced Mucus Secretion in the Porcine Airway Gland

**Figure 3:**
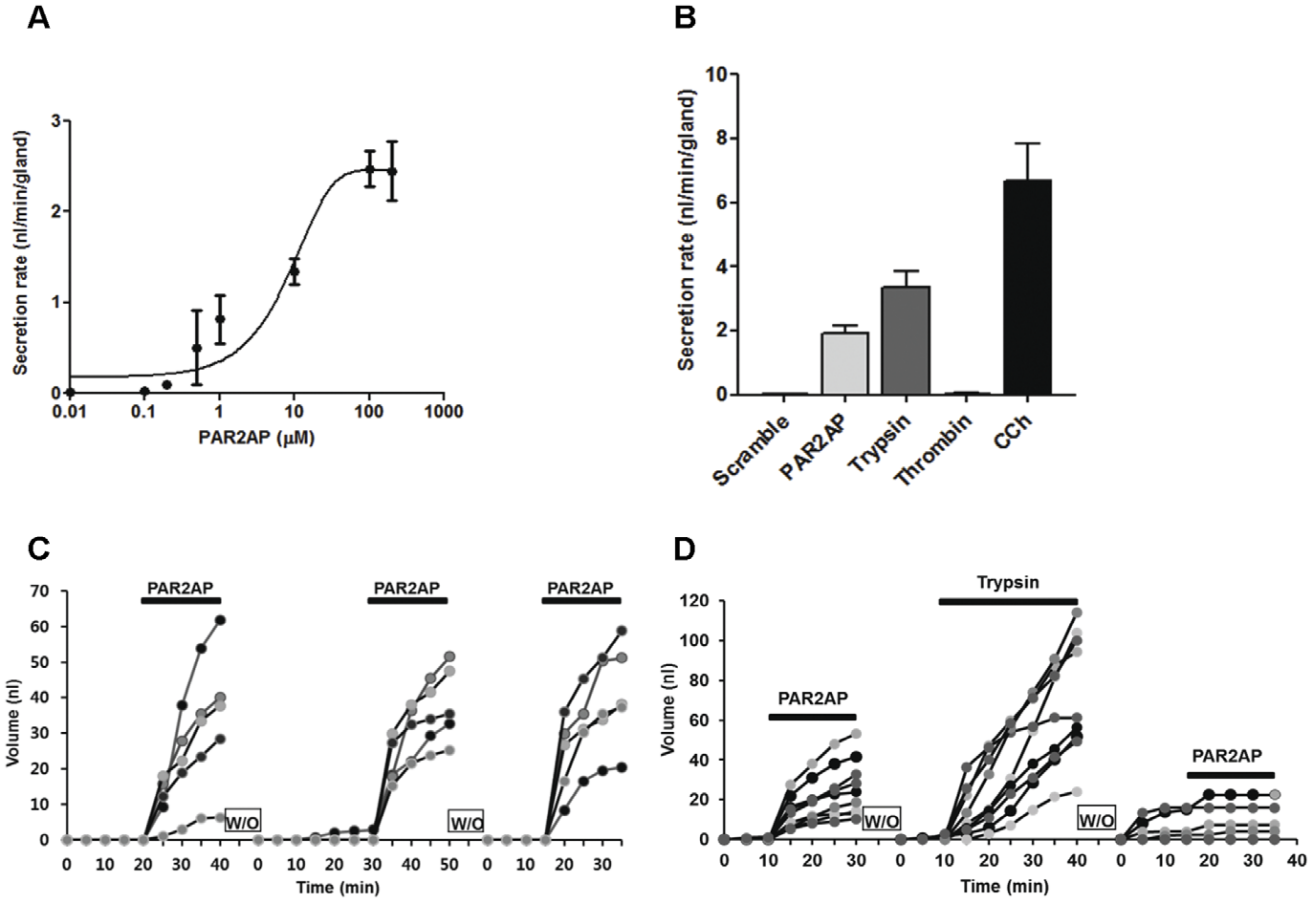
Effects of PAR activators in pig airway gland. (A) Approximate dose-response relationship for mucus secretion in pig submucosal glands. Each point represents the average of 10–14 glands from 3–4 different pig trachea. **(**B) Secretion rates from airway gland in response to PAR activators. Data represent mean ± S.E.M. secretion rates from 11 to 41 glands from two to four pigs. (C) Typical gland response to repeated PAR2-AP application. Similar secretory responses were noted with repeated PAR2-AP treatment. (D) Trypsin (10 µM) induced vigorous mucus secretion, which desensitized the PAR2-AP (10 µM) response.

Because pig airway tissue was more readily available than human tissue, we used pig tracheas to dissect the mechanism of PAR2-mediated mucus secretion. We first established the dose-response relationship for PAR2-AP in the pig airway gland using optical methods to determine the rate of mucus secretion from single glands. The pig gland was more sensitive to PAR2-AP than the human airway gland as the threshold for PAR2-AP stimulation of porcine gland mucus secretion (defined as the concentration that produced an obvious increase in mucus secretion rates for at least two glands in the optical field) was approximately 200 nM, compared to 1 µM in human tissue. The EC_50_ was 12.98 µM, and the approximate *Vmax* achieved with 100 µM PAR2-AP was 2.47±0.2 nl/min/gland (*n* = 3, 27 glands; Figure 3A). The mean secretion rate with PAR2-AP treatment (10 µM) was 1.92±0.2 nl/min, which is approximately 40% of the response achieved with 10 µM carbachol (Figure 3B). Trypsin (10 µM), which can activate PAR2 and possibly PAR4 [20], induced vigorous mucus secretion at a rate of 3.34±0.52 nl/min (41 glands, four pigs). However, thrombin, an activator of PAR1, PAR3, and PAR4, did not induce mucus secretion (Figure 3B). Mucus secretion did not decrease with repeated exposure to PAR2-AP. However, the glandular response to PAR2-AP was almost eliminated after trypsin treatment (Figure 3C and D).

### Lysozyme Concentration of Submucosal Gland Secretion

**Figure 4:**
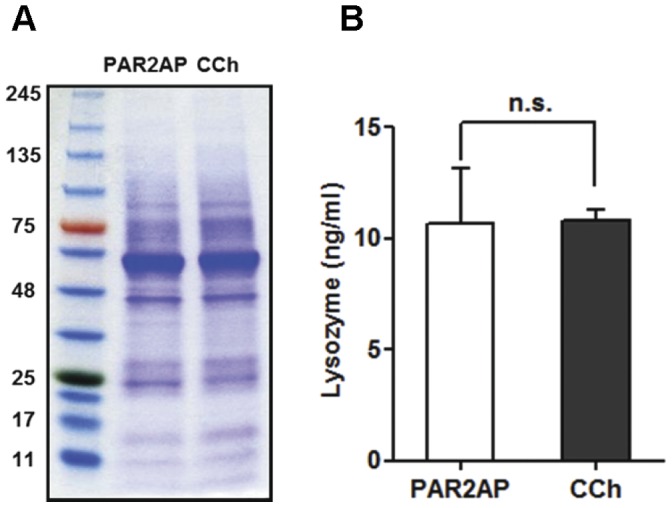
Protein analyses for mucus secretion from porcine airway gland. (A) Coomassie Blue-stained protein bands on a Tris-glycine 4–20% gradient SDS polyacrylamide gel. Mucus secretion was stimulated either by 10 µM PAR2-AP or 10 µM carbachol (CCh). Protein molecular weight standards are shown on the left. (B) Lysozyme concentration in PAR2-AP (10 µM) and carbachol (10 µM)-stimulated mucus from three pigs.

Mucus secretion from porcine tracheas was analyzed on polyacrylamide gels with Coomassie Blue staining (Figure 4A). No differences were found between PAR2AP- or carbachol-induced mucus in either the number or intensities of the bands, indicating that these two methods of stimulation have similar effects on protein secretion by submucosal glands. The lysozyme concentration in PAR2-AP-induced glandular secretion was 10.70±2.42 ng/ml, similar to the concentration of lysozyme in secretions induced by treatment with 10 µM carbachol (10.82±0.47 ng/ml, three pigs; Figure 4B).

### Ca^2+^ and Anion Dependency of PAR2-mediated Mucus Secretion

**Figure 5:**
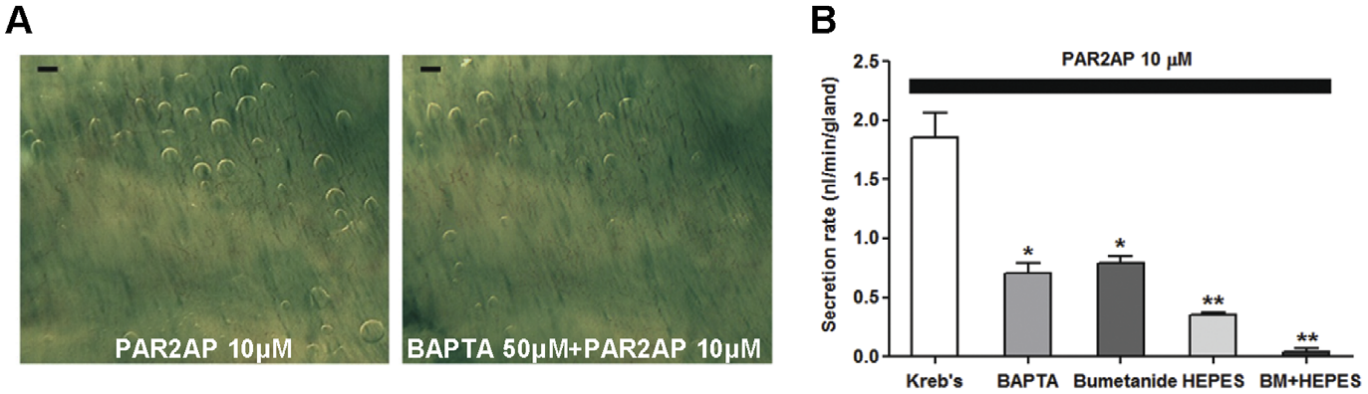
Ca^2+^ and anion dependency of PAR2-AP-induced mucus secretion in pig airway glands. (A) Images of mucus bubbles with and without BAPTA-AM (50 µM). Scale bar: 0.5 mm. (B) Summary data from 21 to 33 glands from three pigs. Data represent mean ± S.E.M. mucus secretion rates. * and ** indicate significant differences from the response seen with PAR2-AP alone, *P*<0.05 and 0.005, respectively.

We compared secretory responses to PAR2-AP in the presence or absence of a Ca^2+^ chelator, BAPTA-AM. BAPTA-AM (50 µM) reduced gland secretion stimulated by PAR2-AP by approximately 50% (0.70±0.08 nl/min, 21 glands, three pigs) compared with the secretion rate measured without BAPTA-AM (1.86±0.20 nl/min; Figure 5A and B). We used bumetanide (100 µM) to block the basolateral Na^+^-K^+^-2Cl^−^ cotransporter 1 (NKCC1) to reduce luminal Cl^–^mediated fluid transport. In a separate experiment, we replaced HCO_3_^−^ in the bath with HEPES and bubbled air through it to eliminate HCO_3_^–^mediated fluid transport. PAR2-AP-stimulated secretion was inhibited by both bumetanide (0.79±0.06 nl/min) and HEPES replacement (0.35±0.02 nl/min) and was nearly eliminated when these treatments were used in combination (0.04±0.02 nl/min; Figure 5B). These results indicate that most PAR2-induced mucus secretion by submucosal glands is dependent on Ca^2+^ mobilization and anion movement.

### PAR2-AP-induced Mucus Secretion in PAR-2 knock out andΔF508 CFTR Mutant Mice

**Figure 6:**
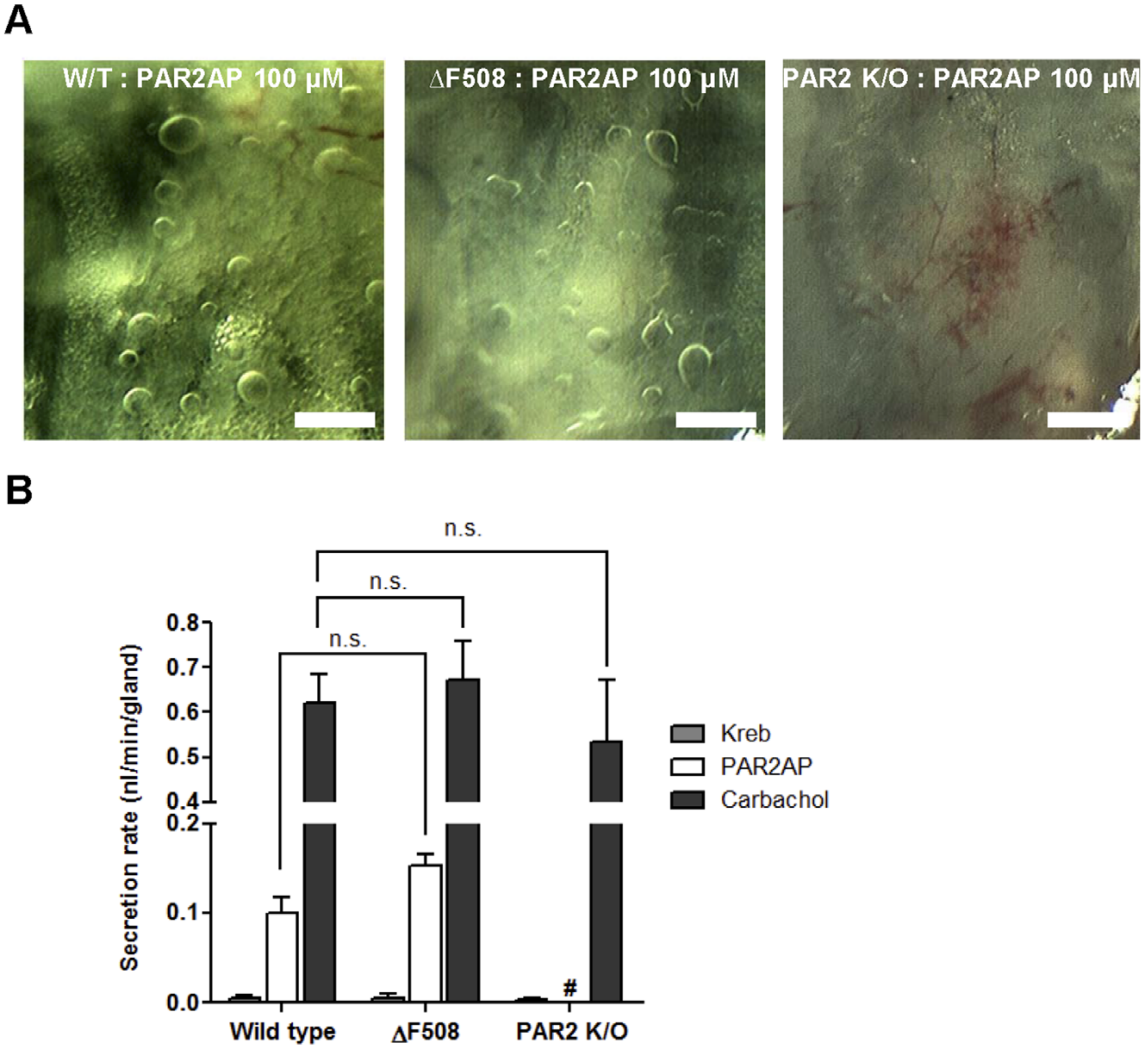
PAR2-AP-induced mucus secretion in mice. (A) Images of mucus bubbles in the tracheas of wild type littermates, PAR-2 knock out and targeted ΔF508 CFTR mutant mice 10 min after PAR2-AP stimulation. Scale bar: 0.5 mm. (B) Secretion rates in response to PAR2-AP (100 µM) or carbachol (10 µM) in mouse tracheal glands. Data represent mean ± S.E.M. secretion rates from four to eight mice.

To examine the possible role of PAR2 and CFTR in PAR2-AP-induced mucus secretion, we compared responses in trachea from PAR-2 knock out and ΔF508 CFTR mutant mice and their wild-type (WT) littermates. When the trachea were exposed to PAR2-AP (100 µM), large amounts of secretion were observed in tracheas from both WT (0.10±0.01 nl/min, 22 glands, four mice) and CF mice (0.15±0.01 nl/min, 15 glands, three mice), with no significant difference between the two groups but there is no secretion from PAR-2 knock out mice (Figure 6A and B).

## Discussion

In previous studies, activation of PAR2 induces a transepithelial anion current in tracheal epithelium, which leads to a shift from absorption to secretion [15], [21]. PAR2 activation by human airway trypsin-like protease in NCI-H292 cells led to a small but significant increase in mucin production, whereas PAR2-AP did not increase mucin secretion [22]. PAR2-AP was also a weak enhancer of mucin secretion in primary human airway epithelial cells [19], which was believed to indicate that PAR2 is not a significant contributor to mucus regulation. However, in the present investigation, we directly demonstrated that PAR2 activation with a synthetic activating peptide, as well as physiological stimulator such as trypsin, significantly induced airway gland mucus secretion. Comparing secretion rates with previous studies in which we used the same methods, the secretion rate induced by PAR2-AP in the human gland was approximately 30% of that induced by carbachol treatment [23] but higher than the responses induced by VIP [2] or substance P [5]. In addition, tiny bubbles were observed on the airway surface after treatment with PAR2, but the bubbles did not become bigger despite continuous stimulation. This suggests that the main target of PAR2 is the submucosal gland and not the airway surface epithelium. Although PAR2 is mainly expressed in serous cells of the airway gland, the protein content and lysozyme concentration of PAR-2 induced mucus does not differ from that of carbachol-induced mucus. Therefore it remains necessary to elucidate whether PAR2-AP stimulated mucus secretion only from serous cells or from other cells as well.

We further dissected the underlying mechanism for PAR2-AP-induced mucus secretion in the airway glands of pigs. Activation of PAR2 elevated intracellular Ca^2+^, and PAR2-AP-induced mucus secretion was decreased by pretreatment with a Ca^2+^ chelator (BAPTA-AM). These results indicate that PAR2-mediated mucus secretion is Ca^2+^-dependent. The response to PAR2-AP was not suppressed by indomethacin (data not shown), and PAR2-AP was still able to induce mucus secretion in the ΔF508 CFTR mutant mouse. These findings are not consistent with a previous report on research performed in Calu-3 cells [16] in which Cl^−^ secretion induced by PAR2-AP was found to require prostaglandin release and CFTR activation. Although Calu-3 cells have properties similar to those of airway gland serous cells, their anion channel profile is quite different from that of the airway gland tissue *in vivo*. That is, the CFTR is the only anion channel in the apical membranes of Calu-3 cells. Thus, in these cells, the apical anion conductance is almost completely derived from CFTR channels [24]. In contrast, both CFTR and CaCC exist in the airway submucosal gland [23]. It is possible that the elevated cytosolic Ca^2+^ induced by PAR2-AP activated the CaCC and thus induced anion secretion in the airway gland *ex vivo* in our experiments. Another discrepancy between Calu-3 cells and airway gland serous cells is that they respond differently to repeated stimulation. Sato *et al.* [17] have shown that PAR2-AP generates a brief response of Cl^−^ secretion through the phosphatidylcholine-phospholipase C (PC-PLC)-mediated pathway in Calu-3 cells, which became desensitized by repeated PAR2-AP treatment. However, we observed that the mucus secretion in response to PAR2-AP did not decrease with repeated treatment in airway glands. A possible reason for this difference is that the turnover rate for PAR2 is more rapid in submucosal gland tissue than in cultured cells.

Although the parasympathetic pathway primarily controls airway gland secretion, evidence increasingly supports a role for intrinsic control systems for airway gland secretion, such as the capsaicin-sensitive C-fiber system [5]. In our experiments, endogenous PAR2 agonists, such as airway trypsin and neutrophil elastase, also stimulated airway mucus secretion from the submucosal gland. This local receptor-mediated mucus secretion may be involved in host defense against pathogens in airway mucosa, which is independent of parasympathetic control. Furthermore, because the PAR2-AP-induced mucus system is independent of the CFTR, this mechanism would be preserved in the airways of patients with CF and could act as a salvaged route for fluid secretion and innate immune responses. We plan to investigate this system in CF patients.

Interestingly, PAR2 expression is increased in airway epithelial cells in allergic airway disease [25] and in bronchial vessels of patients with bronchitis [18]. Furthermore, human airway tryptase (HAT) is detected in high levels in BAL fluid from patients with chronic airway inflammatory disease [20]. Although not shown, our recent data [26] also revealed that PAR2 expression in the airway glands is increased in patients with allergic rhinitis. Thus, PAR2 upregulation may represent an underlying mechanism of mucus hypersecretion in allergic or inflammatory airway disease.

In summary, we demonstrated that PAR2-AP increases mucus secretion from the airway glands of three different species (human, pig, and mouse) and that this effect is Ca^2+^-dependent and at least partially CFTR-independent. If PAR2 is involved in the airway host defense system, future research should focus on the potential of this receptor as a target for therapeutic intervention.

## Materials and Methods

### Chemicals

Fura2-acetoxymethyl ester (fura2-AM) was purchased from Teflabs (Austin, TX, USA). PAR2 activating peptides (PAR2-AP, SLIGRL-NH_2_) and scrambled peptide (LSIGLR-NH_2_) were purchased from the Korea Basic Science Institute (Seoul, Korea). Peptide structure was confirmed by liquid chromatography/mass spectroscopy (HP 1100 series HPLC system, Hewlett Packard, Palo Alto, CA, USA). Trypsin, indomethacin, thrombin, bumetanide, and 1,2-bis(2-aminophenoxy) ethane-N,N,N,N-tetraacetic acid-acetoxymethyl ester (BAPTA-AM) were purchased from Sigma-Aldrich (St. Louis, MO, USA). Collagenase NB 4 was purchased from Serva (Heidelberg, Germany).

### Human Tracheal Tissue

These studies were approved by the Institutional Review Board of Yonsei University College of Medicine (4-2010-0216), and informed consent was obtained from all patients. Small tracheotomy flaps (1 cm^2^) were obtained from the first or second tracheal ring after tracheotomy for airway maintenance in human patients. The subjects (*n* = 11) had no lung disease and their ages ranged from 35 to 72 years (mean = 59.3 years). All tissues were transferred to ice-cold Krebs-Ringer bicarbonate buffer (KRB) bubbled with 95% O_2_–5% CO_2_, in which they were maintained until use, usually within 4 hr. The KRB composition was 115 mM NaCl, 2.4 mM K_2_HPO_4_, 0.4 mM KH_2_PO_4_, 25 mM NaHCO_3_, 1.2 mM MgCl_2_, 1.2 mM CaCl_2_, 10 mM glucose, and 1.0 µM indomethacin. KRB was made to 90% volume, and the osmolarity was measured with a Wescor 5500 vapor pressure osmometer (Logan, UT, USA). Distilled water was added to adjust the osmolarity to 290±5 mOsm. The pH was verified to be 7.4 (Corning Life Sciences, Lowell, MA, USA) after bubbling with 95% O_2_–5% CO_2_.

### Animals

This study was approved by the Committee on Animal Research at Yonsei Medical Center, and all experiments with animals were performed under appropriate guidelines. Pig tracheas were harvested from 18 juvenile Yorkshire pigs of either sex weighing 40–110 kg, following robotic surgery performed for training purposes. CF model mice (*n* = 3) containing the targeted ΔF508 CFTR mutation and WT littermates (*n* = 4) were kindly provided by Dr. M.G. Lee (Yonsei University, Seoul, Korea). PAR-2 knock out mice (*n* = 4) were provided by Dr. M.H Sohn (Yonsei University, Seoul, Korea). The tails were clipped at 18 days of age, and genomic DNA was isolated for subsequent genotyping.

### Optical Measurement of Mucus Secretion Rates (Mucus Bubble Method)

**Figure 7:**
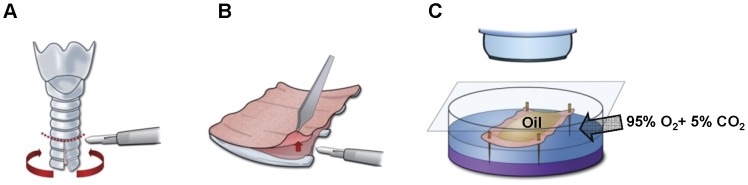
Experimental set-up for measuring mucus secretion from airway gland. (A) A piece of trachea was harvested, cut down the ventral midline and spread out. (B) The cartilage was removed carefully without damaging the submucosal glands. (C) After the tissue was placed so that the luminal surface faced the O_2_-saturated air and the basolateral surface was immersed in KRB solution, the mucosal surface was covered with water-saturated oil.

To prepare tissues for optical recording of mucus secretion rates for individual glands, a tracheal ring of approximately 1.5 cm was cut off and opened up along the ventral midline. The mucosa with underlying glands was dissected from the cartilage and mounted in a 35-mm diameter Sylgard (Dow Corning Corporation, Midland Township, MI, USA)-lined plastic Petri dish with the serosa in the bath (∼1-ml volume of KRB solution) and the mucosa exposed to the air (Figure 7). The tissue chamber was maintained at 35–37°C with high humidity using either a Sensortek S-4 Peltier effect temperature controller and spiral glass humidifier or a thermistor-controlled warming chamber and humidifier (Medical Systems, Greenvale, NY, USA). The tissue surface was cleaned and blotted dry with cotton swabs and further dried with a stream of gas, after which 20–30 µl of water-saturated mineral oil was placed on the surface. The tissue was warmed to 37°C at a rate of approximately 1.5°C/min and continuously superfused with warmed, humidified 95% O_2_–5% CO_2_. Pharmacological agents were diluted to final concentrations with warmed, gassed bath solution, and added to the serosal side of the tissue by complete bath replacement. Bubbles of mucus within the oil layer were visualized by oblique illumination, and digital images were captured with either the macro mode of a Nikon digital camera (Tokyo, Japan) or by mating a digital camera to one ocular of a Wild stereomicroscope. Each image contained an internal reference grid to compensate for any minor adjustments in magnification made during the experiment. Stored images were analyzed either by direct measurement or with ImageJ software (http://rsb.info.nih.gov/ij/). Mucus volumes were calculated from the size of the spherical bubbles and are given as nl/min/gland. Bubbles that were not approximately spherical were omitted from secretion rate analyses. This procedure was performed as described previously [27].

### Measurement of [Ca^2+^]_i_

Human airway submucosal gland tissues (*n = *3) were dissected from tracheotomy flaps, and the tissues were further dissected using collagenase NB4 (Serva, Heidelberg, Germany). The isolated submucosal glands were seeded onto poly-L-lysine (Sigma, St. Louis, MO, USA)-coated glass coverslips (22×22 mm) in 35-mm dishes and cultured for 2 days. Cells were incubated for 1 hr in physiological salt solution (PSS) containing 5 µM fura2-AM in the presence of Pluronic F-127 (Invitrogen, Carlsbad, CA, USA) to enhance dye loading. Fura2-AM-loaded cells were mounted on the stage of an inverted microscope (Nikon) for imaging. The cells were illuminated with light of wavelengths 340 nm and 380 nm, and the emitted fluorescent images at 510 nm were collected with a CCD camera and analyzed using the MetaFluor system (Universal Imaging Co., Downingtown, PA, USA). The fluorescence ratio (340/380) was taken as a measure of [Ca^2+^]_i_, and fluorescence images were obtained at 3-sec intervals.

### Immunostaining and Evaluation of PAR2 Staining

Tracheal mucosa harvested during tracheotomies in patients with intracranial hemorrhage were fixed with 10% formaldehyde solution for 24 h and then dehydrated and embedded in paraffin. Paraffin blocks were sectioned into 4-µm-thick slices. After deparaffinizing and rehydrating, slides were incubated in antigen retrieval solution (Tris-EDTA, pH 9.0) for 20 min at 95–100°C. To block endogenous peroxidase, slides were treated with 0.3% H_2_O_2_ for 15 min at room temperature. Slides were blocked in 10% normal serum with 1% BSA in TBS for 2 h at room temperature and then incubated overnight at 4°C with a monoclonal mouse antibody against human PAR2 (1∶100, Santa Cruz Biotechnology, Santa Cruz, CA, USA). The slides were then incubated with horseradish peroxidase-conjugated goat anti-mouse IgG (1∶200; Jackson ImmunoResearch Laboratories, West Grove, PA, USA) in antibody diluent solution (DAKO, Glostrup, Denmark) for 1 hr at room temperature. Slides were developed with DAB (DAKO) at room temperature and counterstained with hematoxylin (Merck KGaA, Darmstadt, Germany).

The extent of the PAR-2 staining, defined as the percentage of positive acinar staining areas, were calculated by manually tracing out the total acinar cell area and PAR-2 positive staining area at 400X magnification using Image J software (http://rsb.info.nih.gov/ij/).

### Coomassie Blue Staining

After application of PAR2AP (10 µM) or Carbachol (10 µM), secreted mucus bubbles were collected by using fine forcep and micro pipette without approaching the epithelium of porcine trachea tissue. Collected mucus were stored at −20°C until use. Oil were removed by centrifugation at 14,000 rpm for 5 min, then add lysis buffer with complete protease inhibitor mixture (Roche Applied Science). After lysis, cleared lysates (30 µg of protein) were separated by 4–20% pre-made gradient SDS-PAGE, then stainined by using coomassie brilliant blue R-250 (sigma) solution.

### ELISA for Lysozyme

Lysozyme level was measured by sandwich enzyme-linked immunosorbent assay (ELISA) (ALPO Diagnostics, Windham, NH). PAR2AP - or Carbachol – stimulated mucus secretion was assayed in duplicate. After appropriate dilution, each sample was carried out as the manufacturer’s direction. Briefly, microtiter strips were washed five times with wash buffer, then add 100 µl of standard, sample, controls in duplicate into respective well. The plate was incubated for 1 hr at room temperature on horizontal mixer. After 5 times washes, 100 µl of conjugate (peroxidase–labeled rabbit– anti–lysozyme) was added into each well and then incubated for 1 hr at room temperature. After 5 times washes, the plate was incubated with the 100 µl of 3,3_,5,5_-tetramethylbenzidine (TMB) substrate for 20 min at room temperature in the dark. Absorbance at 450 nm was recorded using an automated ELISA microplate reader (Molecular Devices).

### Statistical Analysis

Data shown are means ± SEM, and the Student t-test for unpaired data was used to compare the means of different treatment groups unless otherwise indicated. The difference between two means was considered to be significant when *P*<0.05. Curves were fit with Origin software (OriginLab Corporation, Northampton, MA, USA) using a sigmoid function.
